# Supplementary material for: Polyphosphate as a novel regulator of super-enhancer complexes: disruption of phase separation and gene expression
Source: Nucleic Acids Res. 2026 May 26;54(10):gkag530. doi: 10.1093/nar/gkag530 (PMC13202173; doi:10.1093/nar/gkag530)
Supplement: gkag530_Supplemental_Files [file gkag530_supplemental_files.zip › 3. Final_SI_NAR-03690-X-2025.docx]

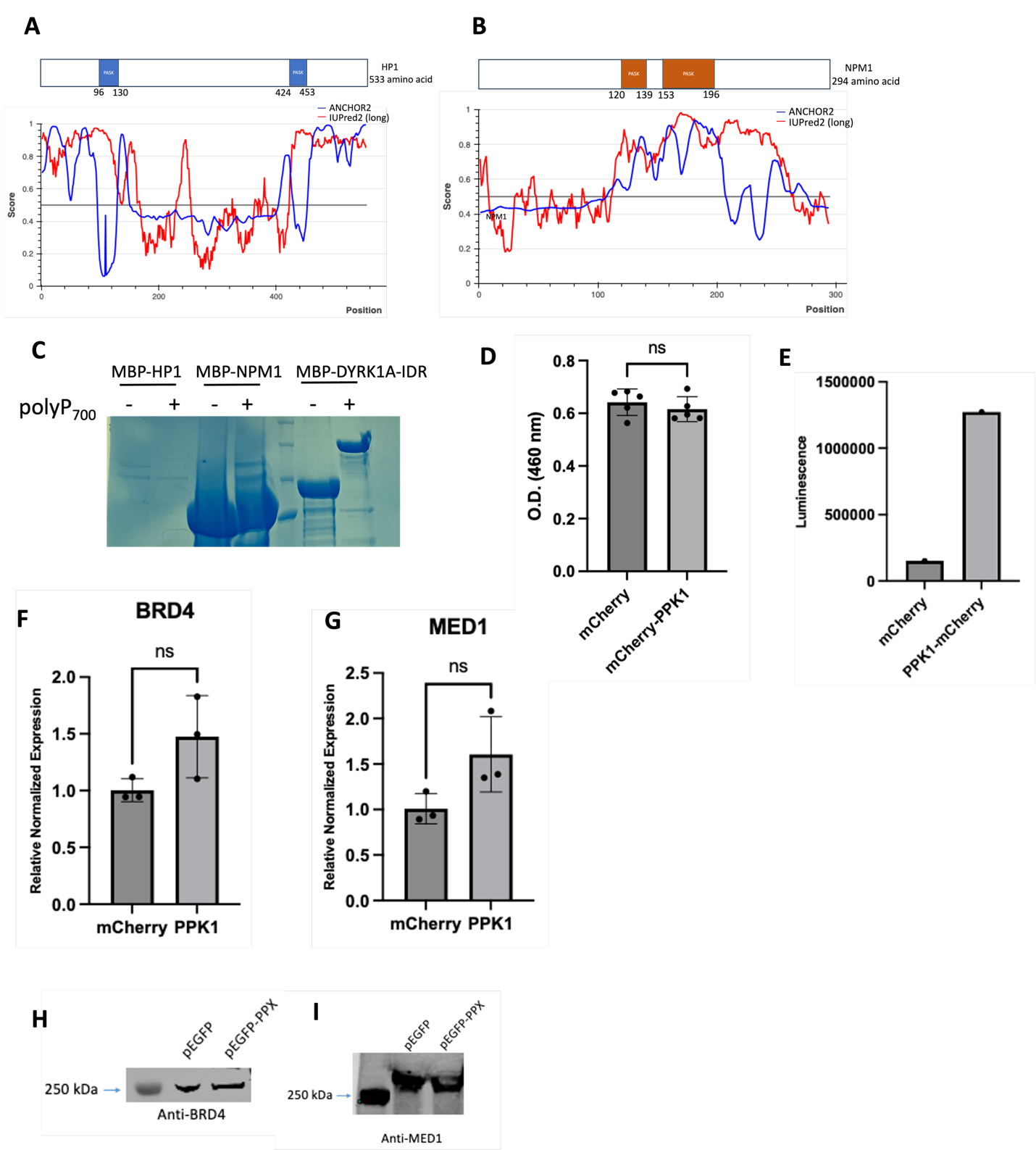


**Figure S1.** Polyphosphate cannot modify HP1 and NPM1. (**A, B**) Domain graphs of HP1 (**A**) and NPM1 (**B**) PASK domains, IDRs prediction based on ANCHOR2 and IUPred2 algorithms. Scores >0.5 indicate disorder. (**C**) Coomassie-stained NuPAGE analysis showing polyphosphate-mediated shift of purified MBP-tagged HP1 and NPM1 with polyP_700_. (**D**) Cell viability of HeLa cells after transfected with mCherry or mCherry-PPK1 were analyzed using WST-8 assay. (**E**) Quantification of polyP. PolyP extractions from HeLa cells transfected with mCherry or mCherry-PPK1 were quantified by luciferase assay using ATP determination kit. (**F, G**) Quantification results of mRNA expression levels of *BRD4* (**F**) and *MED1* (**G**) genes in HeLa cells transfected with plasmids expressing either mCherry or mCherry-PPK1. Unpaired t test; ns, p > 0.05, ^∗∗^p ≤ 0.01, ^∗∗∗∗^p ≤ 0.0001, error bars ± standard deviation. (**H, I**) PolyP modification of BRD4 (**H**) and MED1 (**I**). HeLa cells transfected with pEGFP or pEGFP-PPX were analyzed via NuPAGE followed by western blot with antibodies against BRD4 (**H**) and MED1 (**I**). Images are representative of n = 3.


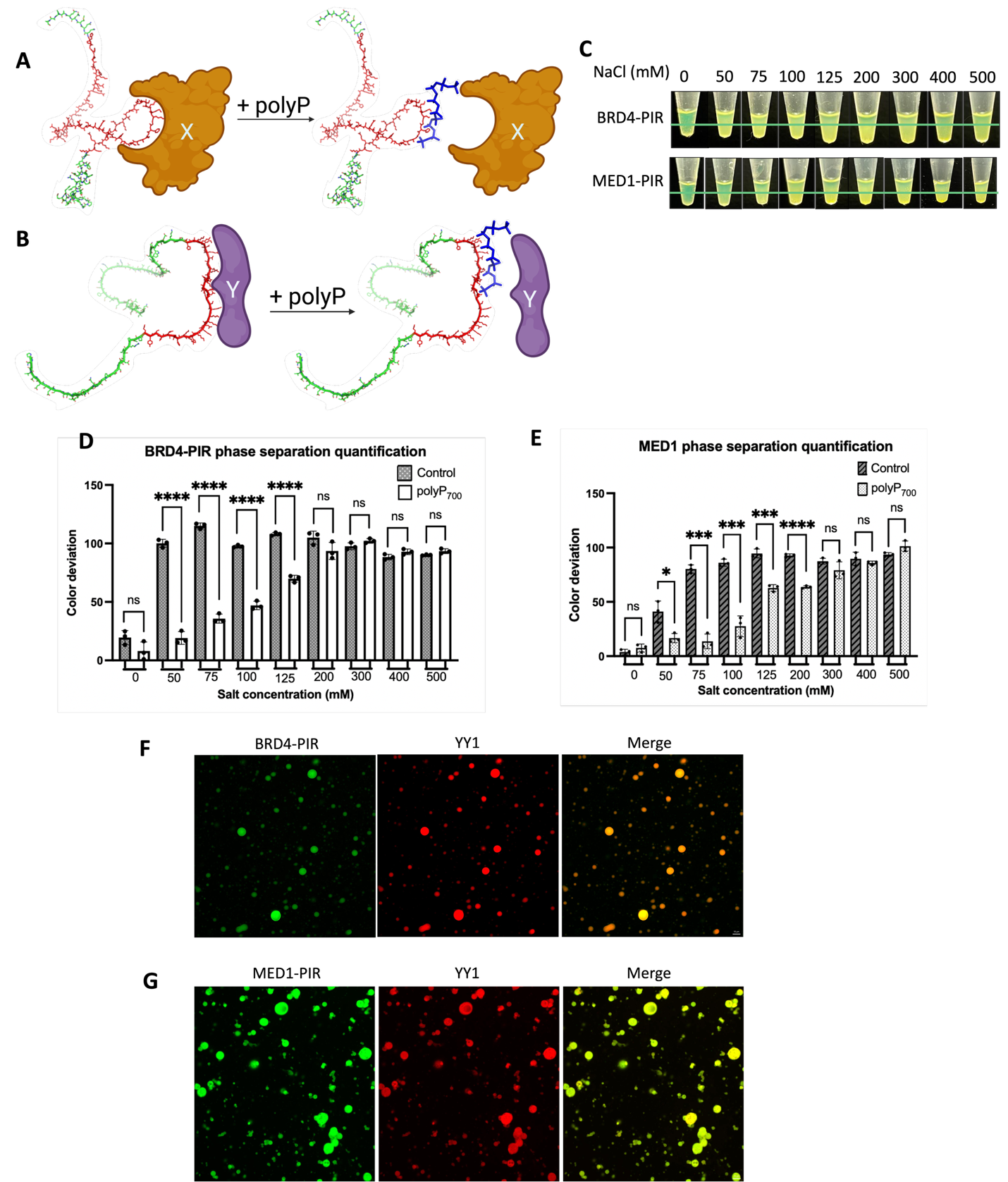


**Figure S2.** Polyphosphate binds to the PIRs of BRD4 and MED1 and disrupts their liquid-liquid phase separation. (**A**) The proposed model showing that polyP prevents the binding of BRD4 to its protein-binding partner (protein X) through PIR enriched in lysine. The PIR (amino acids 648-752) of BRD4 is highlighted in red. Created in BioRender. Jin, J. (2026) <https://BioRender.com/r07g687>. (**B**) The proposed model showing that polyP prevents the binding of MED1 to its protein-binding partner (protein Y) through PIR enriched in lysine. The PIR (amino acids 1458-1581) of BRD4 is highlighted in red. Created in BioRender. Jin, J. (2026) <https://BioRender.com/q96h587>. (**C**) Phase separation of purified MBP-GFP tagged BRD4-PIR and MED1-PIR with indicated concentration of salt. Tubes containing MBP-GFP tagged BRD4-PIR and MED1-PIR in the buffer containing PEG8000 (n=3). (**D, E**) Comparison of color intensity between control and experimental solutions for phase separation of purified MBP-GFP tagged BRD4-PIR and MED1-PIR with and without polyP_700_ under the indicated concentration of salt. (**F, G**) The ability of YY1 droplets to incorporate BRD4-PIR (**F**) or MED-PIR (**G**) proteins *in vitro*. The indicated MBP-GFP or MBP-mCherry fusion proteins were mixed in buffer containing PEG8000 and 100 mM NaCl. Indicated fluorescence channels are presented for each mixture (n=3).


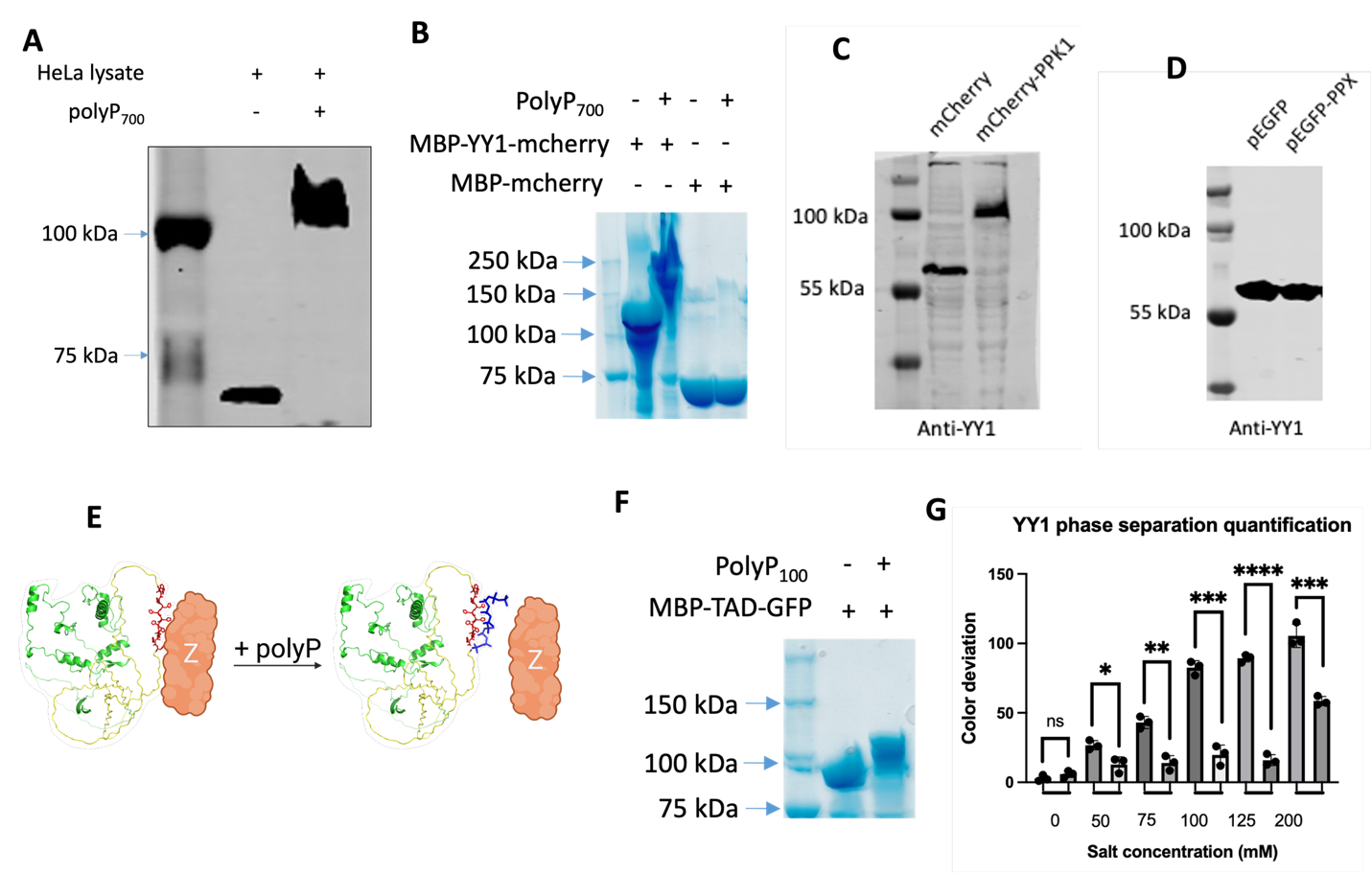


**Figure S3.** Polyphosphate modifies TAD of YY1 through histidine-rich sequence. (**A**) PolyP modification of YY1. HeLa cells lysate with/without the addition of polyP_700_ were analyzed via NuPAGE followed by western blot with antibodies against YY1. (**B**) Coomassie-stained NuPAGE analysis showing polyphosphorylation shift of purified MBP-mCherry tagged YY1 with polyP_700_. **(C, D)** PolyP modification of YY1. HeLa cells transfected with mCherry or mCherry-PPK1 (**C**); and HeLa cells transfected with pEGFP or pEGFP-PPX (**D**) were analyzed via NuPAGE followed by western blot with antibodies against YY1. Images are representative of n = 3. (**E**) The proposed model showing that polyP prevents the binding of YY1 to its protein-binding partner (protein Z) through PIR enriched in histidine. The histidine residues (amino acids 70-80) of YY1 are highlighted in red and YY1’s transactivation domain (amino acids 1-154) is colored in yellow. Created in BioRender. Jin, J. (2026) <https://BioRender.com/q51e851>. (**F**) Coomassie-stained NuPAGE analysis showing polyphosphorylation shift of purified MBP-GFP tagged TAD of YY1 with polyP_100_. (**G**) Comparison of color intensity between control and experimental solutions for phase separation of purified MBP-mCherry tagged YY1 with and without polyP_700_ under the indicated concentration of salt.


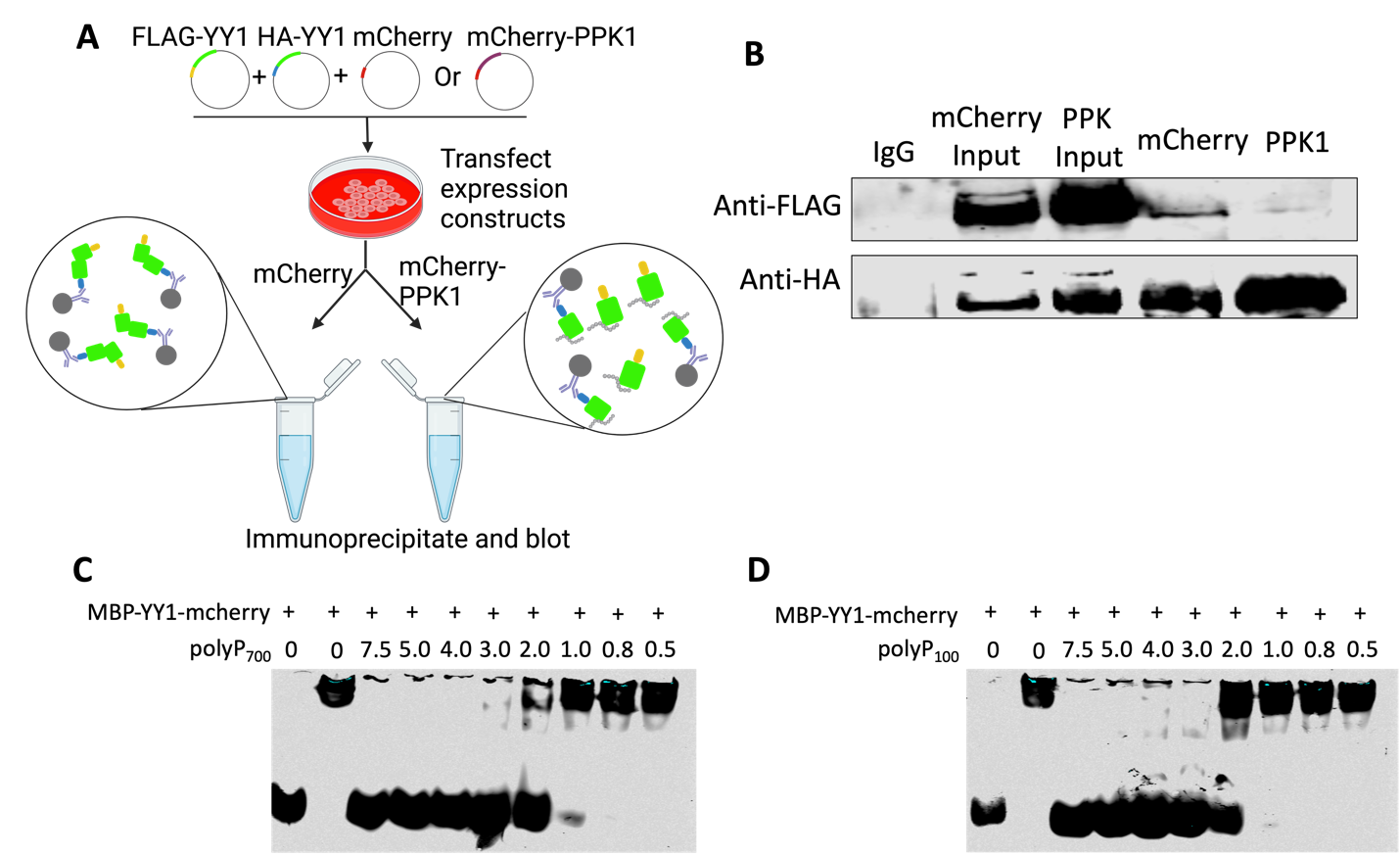


**Figure S4.** Polyphosphate impacts on YY1 functions**.** (**A**) A schematic depicting co-immunoprecipitation assay to detect YY1 dimerization in HeLa cells triple-transfected with FLAG-tagged YY1, HA-tagged YY1 and either mCherry or mCherry-PPK1 using HA antibody. Created in BioRender. Jin, J. (2026) <https://BioRender.com/u19o161>. (**B**) Western blot analysis after SDS-PAGE showing the ability of polyP overproduction in HeLa cells to destroy co-immunoprecipitation of FLAG-tagged YY1 and HA-tagged YY1 proteins from nuclear lysates prepared from transfected cells using antibodies against FLAG or HA (n=3).

(**C, D**) EMSA results using 4% native gel showing the binding of MBP-mCherry tagged YY1 to IR700Dye-labled human telomere G4 structure after treatment with the indicated concentration of polyP_700_ (**C**) and polyP_100_ (**D**).


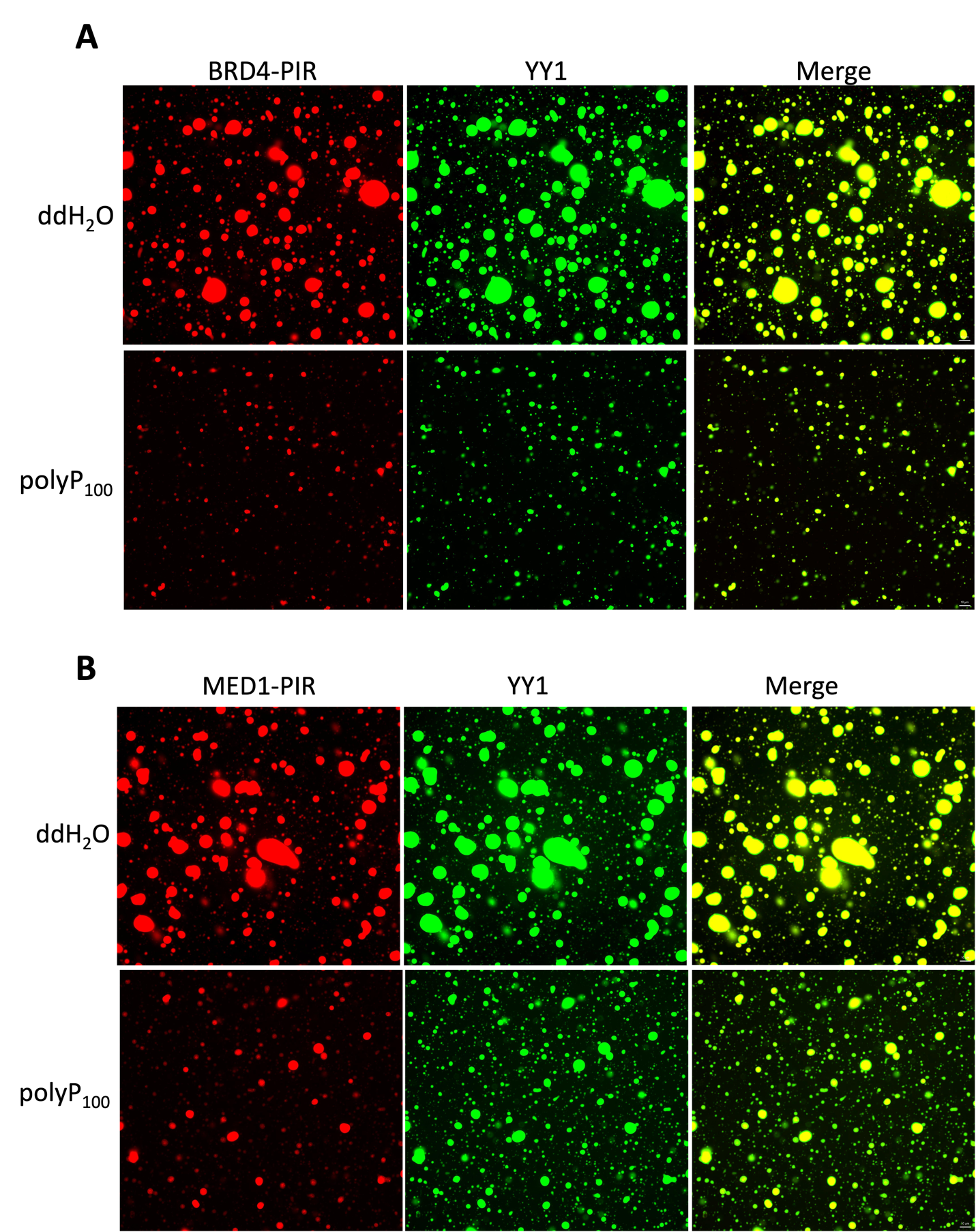


**Figure S5.** Medium chain of polyphosphate impacts the phase separation of BRD4-PIR and MED1-PIR. (**A, B**) PolyP_100_ affects the droplet formation of BRD4-PIR (**A**), MED1-PIR (**B**) and YY1 (**A and B**); but not the ability of YY1 droplets to incorporate BRD4-PIR (**A**) or MED-PIR (**B**) *in vitro*. The indicated MBP-GFP or MBP-mCherry fusion proteins were mixed in buffer containing PEG8000 and 100 mM NaCl. Indicated fluorescence channels are presented for each mixture (n=3).

**
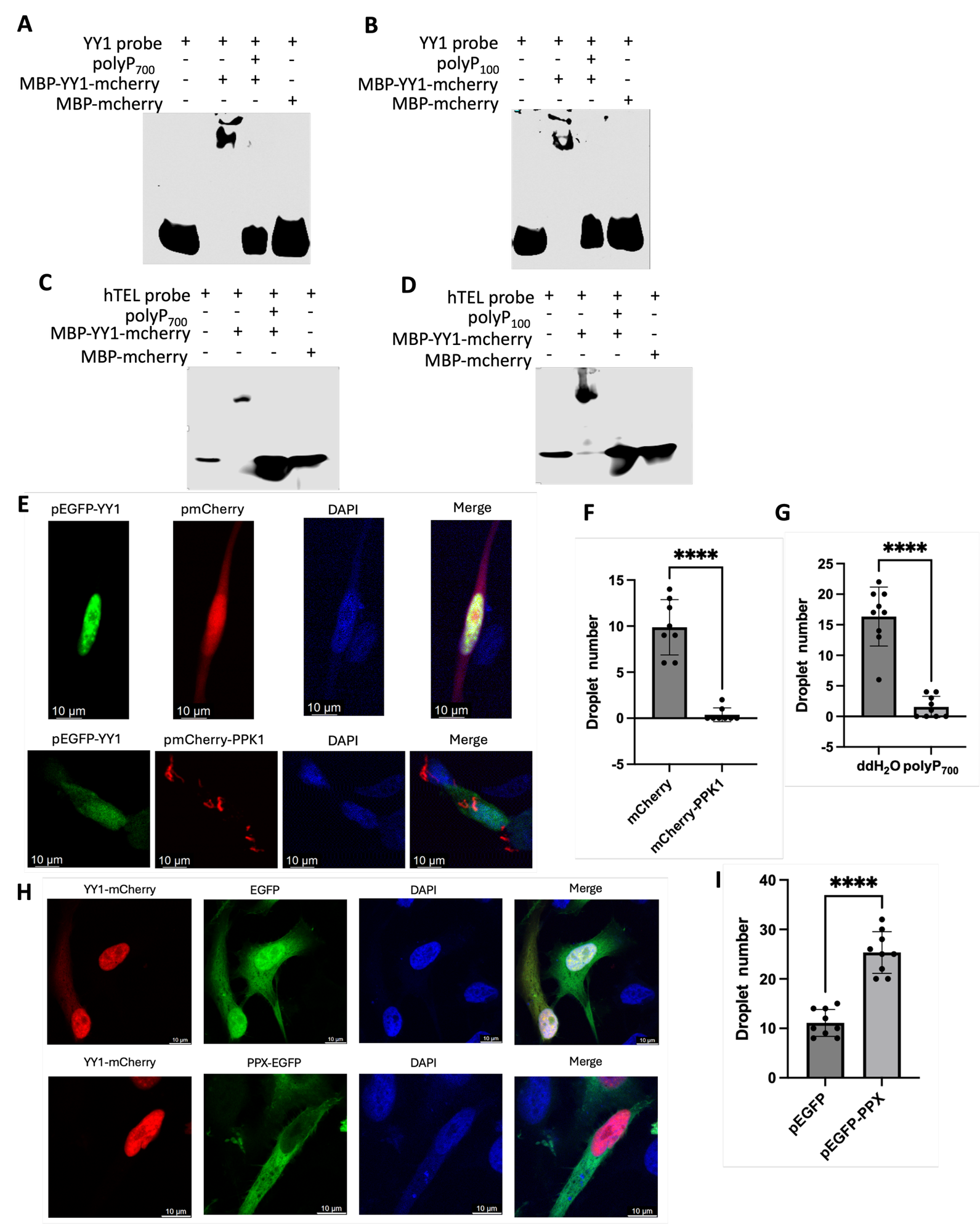
**

**Figure S6.** Replicates of EMSA and YY1 nuclear speckle formation. (**A, B, C, D**) EMSA results using 4% native gel showing the binding of MBP-mCherry tagged YY1 to IR700Dye-labled YY1 binding motif (**A, B**) or IR700Dye-labled human telomere G4 structure (**C, D**) after polyP_100_ or polyP_700_ treatment. (**E, F**) YY1 nuclear speckle formation in HeLa cells were visualized by Confocal microscopy. HeLa cells were co-transfected with GFP-YY1 (first column, green) and either mCherry or mCherry-PPK1 (second column, red), followed by DAPI staining (third column, blue) using ProLong Diamond Antifade Mountant with DAPI. (**G**) Quantification of YY1 droplets formation in HeLa cells. PolyP was added to the fixed GFP-YY1 expressing (first column, green) HeLa cells, followed by DAPI staining (second column, blue). (**H, I**) YY1 nuclear speckle formation in HeLa cells were visualized by Confocal microscopy. HeLa cells were co-transfected with mCherry-YY1 (first column, red) and either pEGFP or pEGFP-PPX (second column, green), followed by DAPI staining (third column, blue) using ProLong Diamond Antifade Mountant with DAPI.

**Table S1.** Oligonucleotide primers used in this study. Relates to Methods.

| Name | Sequence (5’ to 3’) |
| --- | --- |
| mCherry-PPK1-F | ATATAAGCTTCGCCACCATGAATACGCAGCAAGGACTTG |
| mCherry-PPK1-R | ATATGGTACCGCACGTGCGGTAAGCACCG |
| BRD4-PIR-GFP-F | ATATGGATCCATGAACCCCGACGAGATTGAAATCGACTTT |
| BRD4-PIR-GFP-R | ATATGAATTCCACAGGAGCCGGGGCCTGCTG |
| MED1-PIR-GFP-F | ATATGGATCCATGCAGAATCTGGACAGTGAAAGTGAGTCAGGC |
| MED1-PIR-GFP-R | ATATGAATTCATTCCCAATCAGGGCCACATCCATAAG |
| YY1-mcherry-F | ATATGGATCCATGGCGTCTGGTGACACCCTGTAC |
| YY1-mcherry-R | ATATCTCGAGCTGGTTGTTTTTCGCTTTCGCGTGG |
| YY1-TAD-GFP-F | ATATGGATCCATGGCGTCTGGTGACACCCTGTAC |
| YY1-TAD-GFP-R | ATATCTCGAGAGCAACGGTCACCAGGGTCTG |
| MANF-F | GGCGACTGCGAAGTTTGTAT |
| MANF-R | TTTGGTGGCTGCATCATCTG |
| PDHB-F | TCGAGGGCTGTGGAAGAAAT |
| PDHB-R | AATTCACAAATGGGCCGCAA |
| MYC-F | CTCCTACGTTGCGGTCACAC |
| MYC-R | CCGGGTCGCAGATGAAACTC |
| GAPDH-F | TTCGACAGTCAGCCGCATCTTCTT |
| GAPDH-R | CAGGCGCCCAATACGACCAAATC |

**Table S2.** Plasmids used in this study. Relates to Methods

| Insert gene | MCS/Tag | Backbone vector | Description | Source |
| --- | --- | --- | --- | --- |
| mCherry | N-MBP-TEV-insert-mCherry-C | pET16b | Control vector for expressing mCherry | Jia lab |
| GFP | N-MBP-TEV-insert-GFP-C | pET16b | Control vector for expressing GFP | Jia lab |
| YY1 | N-EGFP-insert-C | pEGFP-C1 | Human cDNA-derived *YY1* gene fusion to EGFP | Dr. Susana de la Luna |
| YY1 | N-insert-mCherry-C | pmCherry-N1 | Human cDNA-derived *YY1* gene fusion to mCherry | Jia lab |
| PA PPK1 | N-insert-mCherry-C | pmCherry-N1 | *P. aeruginosa* PA14 *ppk1* gene fusion to mCherry | Jia lab |
| PA PPX | N-EGFP-insert-C | pEGFP-C1 | *P. aeruginosa* PA14 *ppx* gene fusion to EGFP | Jia lab |
| YY1 | N-MBP-TEV-insert-mCherry-C | pET16b | Human cDNA-derived *YY1* gene fusion to mCherry | This study |
| MED1-PIR | N-MBP-TEV-insert-GFP-C | pET16b | MED1 residues 1459-1581 from human cDNA-derived *MED1* gene fusion to GFP | This study |
| BRD4-PIR | N-MBP-TEV-insert-GFP-C | pET16b | BRD4 residues 648-752 from human cDNA-derived *BRD4* gene fusion to GFP | This study |
| YY1 | N-HA-insert-C | pcDNA3 | Human cDNA-derived *YY1* gene fusion to HA | Dr. Richard A. Young lab |
| YY1 | N-FLAG-insert-C | pcDNA3 | Human cDNA-derived *YY1* gene fusion to FLAG | Dr. Richard A. Young lab |
| YY1 binding sites | N/A | pAW49.pUC19 | N/A | Dr. Richard A. Young lab |
| Filler DNA instead of the YY1 motif | N/A | pAW79.pUC19 | N/A | Dr. Richard A. Young lab |
