# Supplementary material for: Polyphosphate as a novel regulator of super-enhancer complexes: disruption of phase separation and gene expression
Source: Nucleic Acids Res. 2026 May 26;54(10):gkag530. doi: 10.1093/nar/gkag530 (PMC13202173; doi:10.1093/nar/gkag530)
Supplement: gkag530_Supplemental_Files [file gkag530_supplemental_files.zip › 4. Final-Additional Data-Original Gel Images-NAR-03690-X-2025.docx]

**
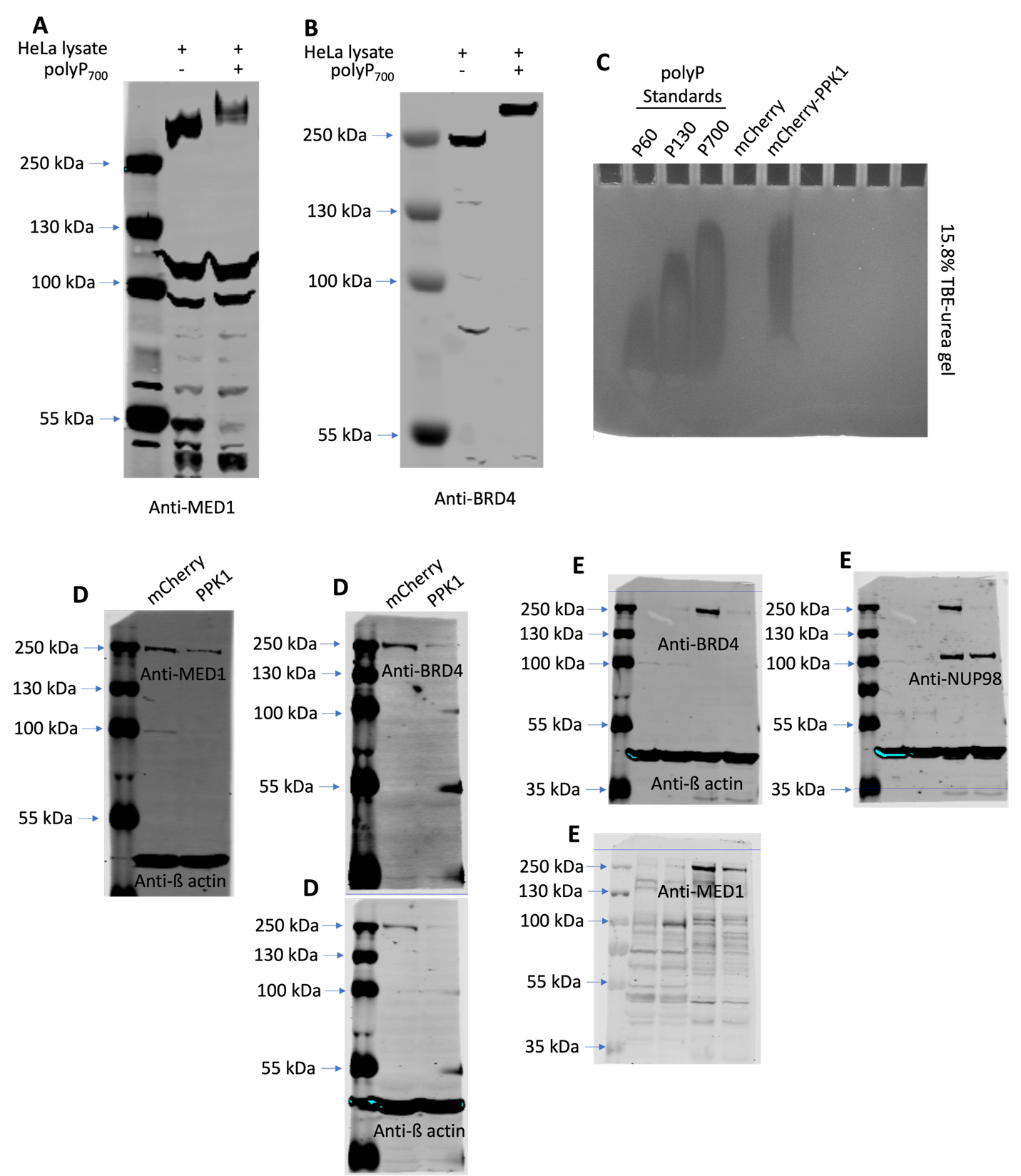
**

**Figure A1.** Original gels or images of Figure 1. (**A,** **B**) Corresponding to Figure 1A and 1B. PolyP modification of BRD4 (**A**) and MED1 (**B**). HeLa cells lysate with/without the addition of polyP_700_ were analyzed via NuPAGE followed by western blot with antibodies against BRD4 (**A**) and MED1 (**B**). Images are representative of n = 3. PolyP_700_ was present at 5 mM unless otherwise indicated. (**C**) Corresponding to Figure 1C. PolyP overproduction in HeLa cells. PolyP extractions from HeLa cells transfected with mCherry or mCherry-PPK1 were analyzed on a 15.8% Tris-borate-EDTA (TBE)-urea gel stained with DAPI. PolyP standards (60, 130 and 700 phosphates) are presented for comparison (n = 3). (**D, E**) Corresponding to Figure 1E and 1F. Western blot analysis after SDS-PAGE of fractions from mCherry or mCherry-PPK1 expressing HeLa cells with antibodies against total proteins (**D**) or proteins localized to specific fractions (**E**): cytoplasm (Cy) and nuclear (Nu). Western blot shown is a representative image of n = 3.

**
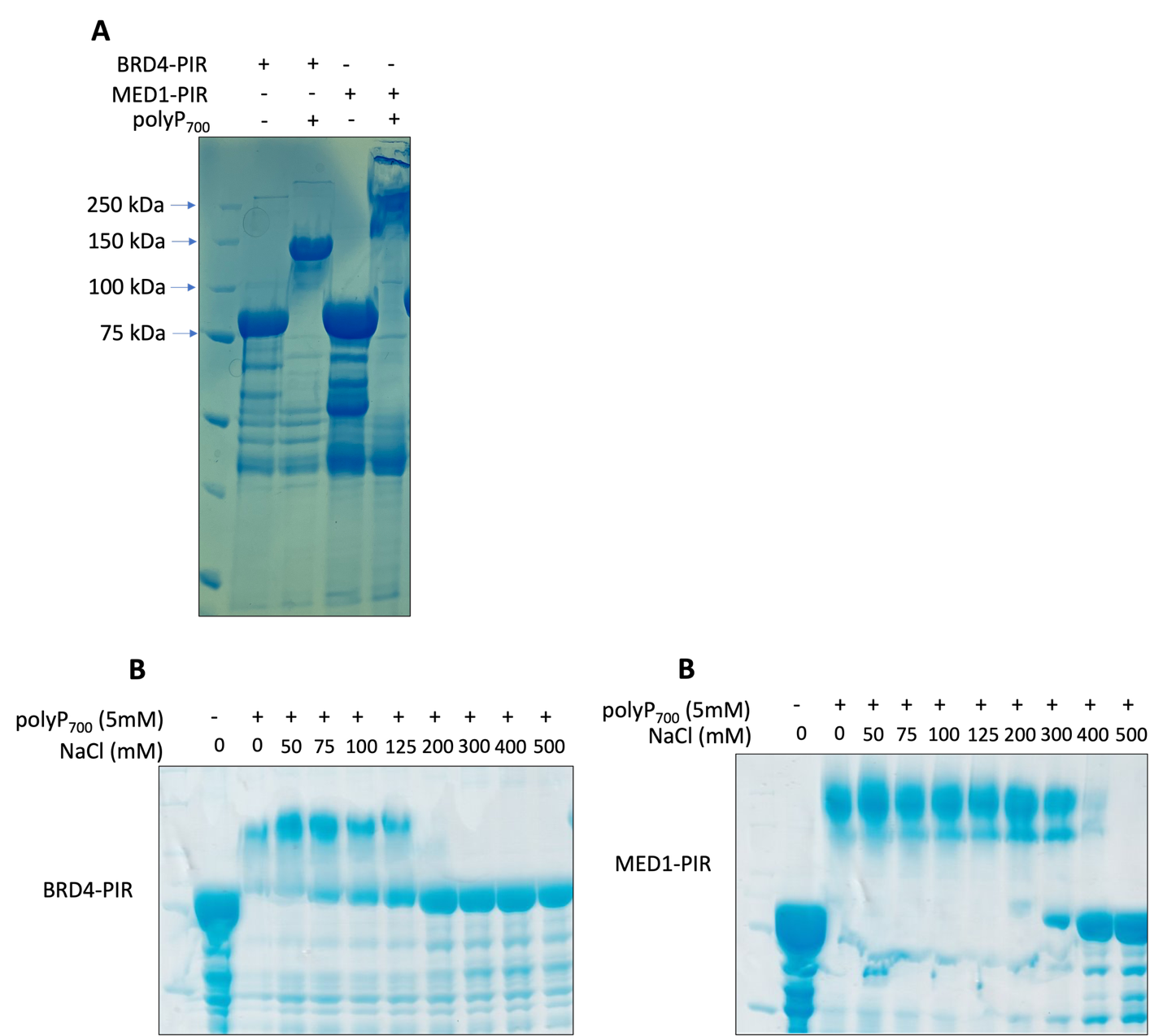
**

**Figure A2.** Original gels of Figure 2. (**A**) Corresponding to Figure 2C. Coomassie-stained NuPAGE analysis showing polyP-mediated shift of purified MBP-GFP tagged BRD4-PIR and MED1-PIR. (**B**) Corresponding to Figure 2D. Coomassie-stained NuPAGE analysis of polyP_700_ modification of purified MBP-GFP tagged BRD4-PIR (Left) and MED1-PIR (Right) with the indicated concentration of salt.

**
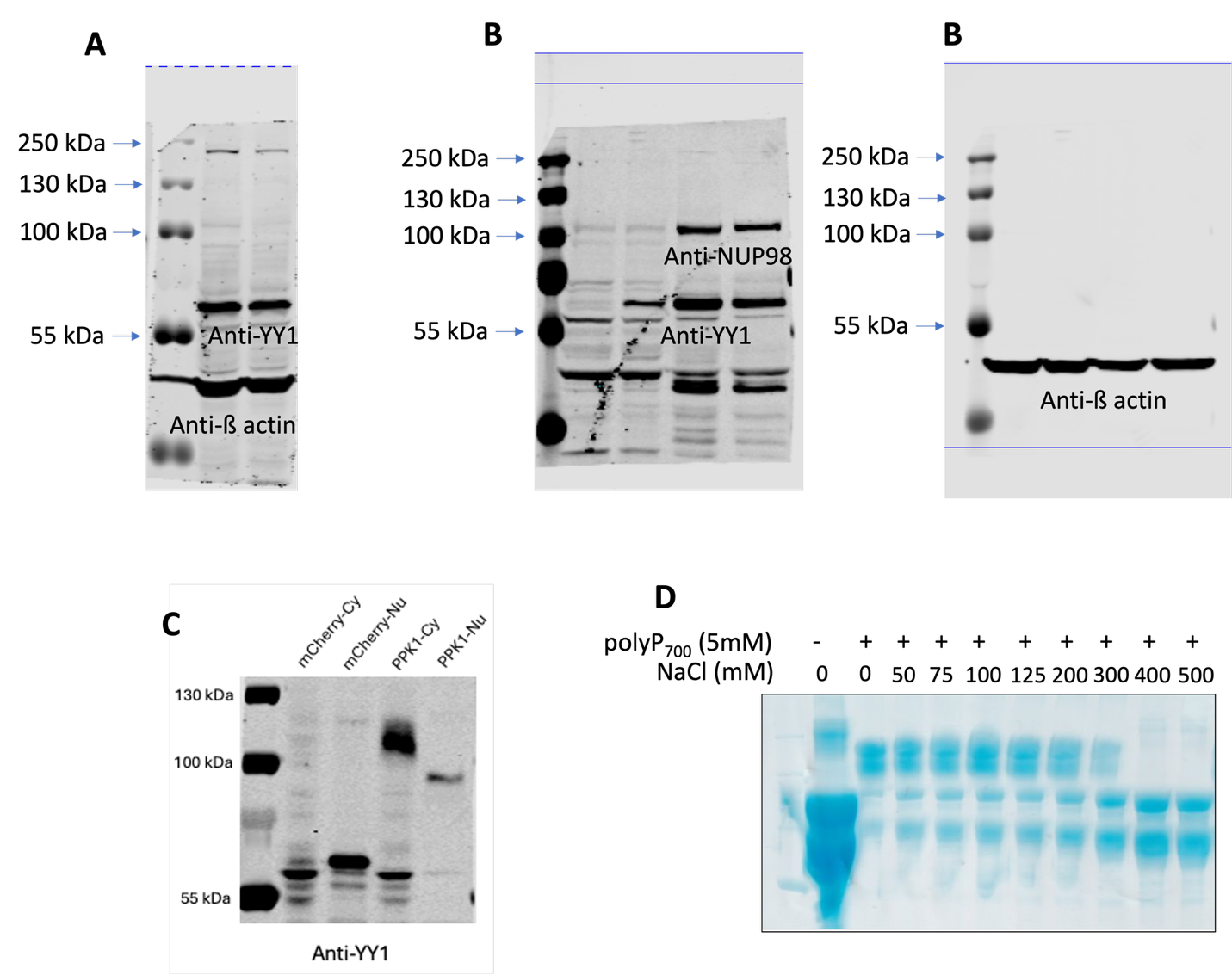
**

**Figure A3.** Original gels or images of Figure 3. (**A**) Corresponding to Figure 3C. Western blot analysis after SDS-PAGE using the YY1 antibody with β-actin as a loading control (n = 3). (**B**) Corresponding to Figure 3D. Western blot analysis after SDS-PAGE of fractions from mCherry or mCherry-PPK1 expressing HeLa cells with antibodies against proteins localized to specific fractions: cytoplasm (Cy) and nuclear (Nu). (**C**) Corresponding to Figure 3F. Western blot analysis after NuPAGE using the YY1 antibody. (**D**) Corresponding to Figure 3J. Coomassie-stained NuPAGE analysis of polyP_700_ modification of purified MBP-m Cherry tagged YY1 with the indicated concentration of salt.

**
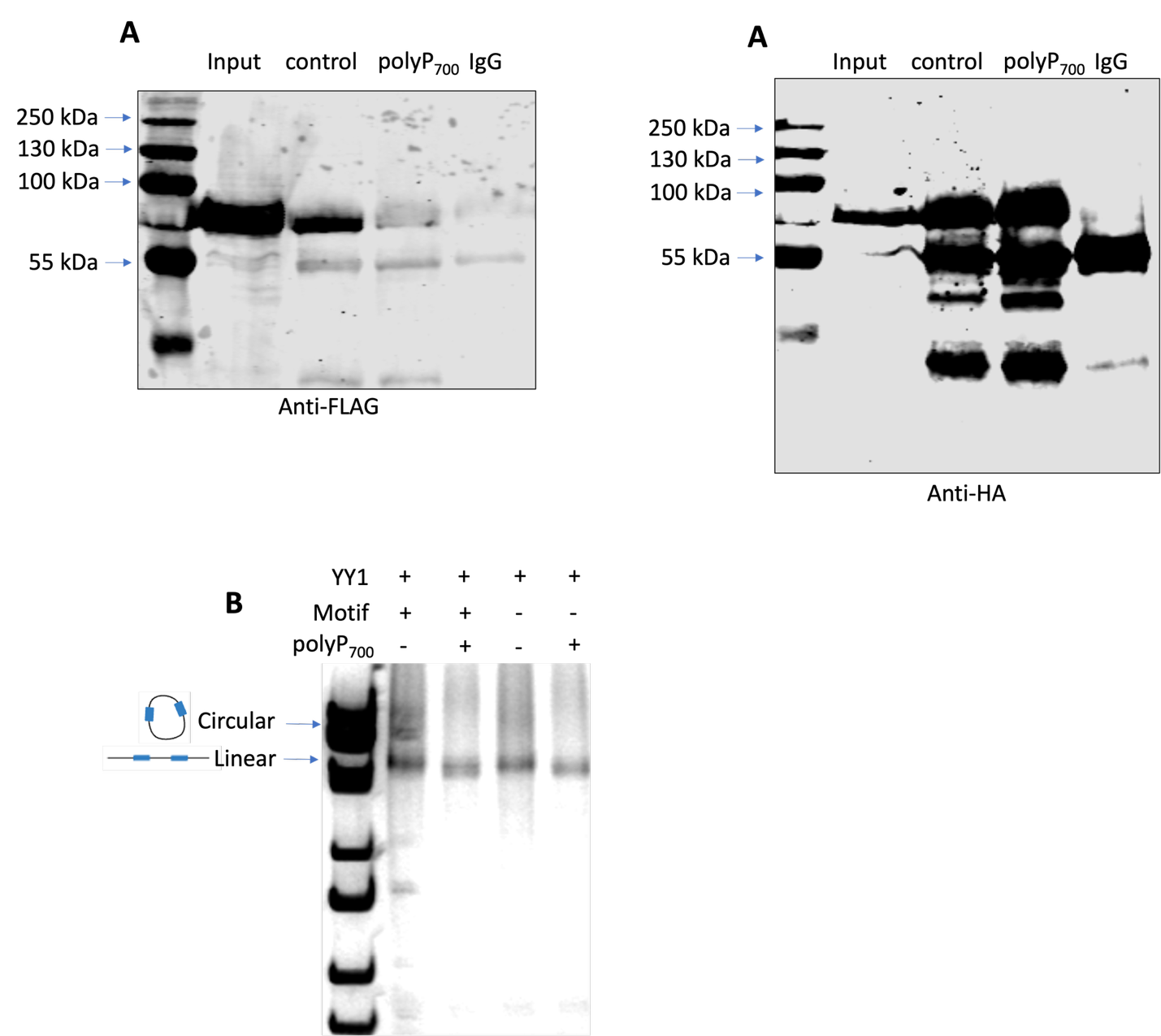
**

**Figure A4.** Original gels or images of Figure 4. (**A**) Corresponding to Figure 4D. Western blot analysis after SDS-PAGE showing the ability of polyP_700_ to disrupt co-immunoprecipitation of FLAG-tagged YY1 and HA-tagged YY1 proteins from nuclear lysates prepared from transfected cells using antibodies against FLAG or HA. (**B**) Corresponding to Figure 4F. Results of the *in vitro* DNA circularization assay visualized by gel electrophoresis showing the ability of polyP_700_ to disrupt YY1-mediated DNA loop formation.

**
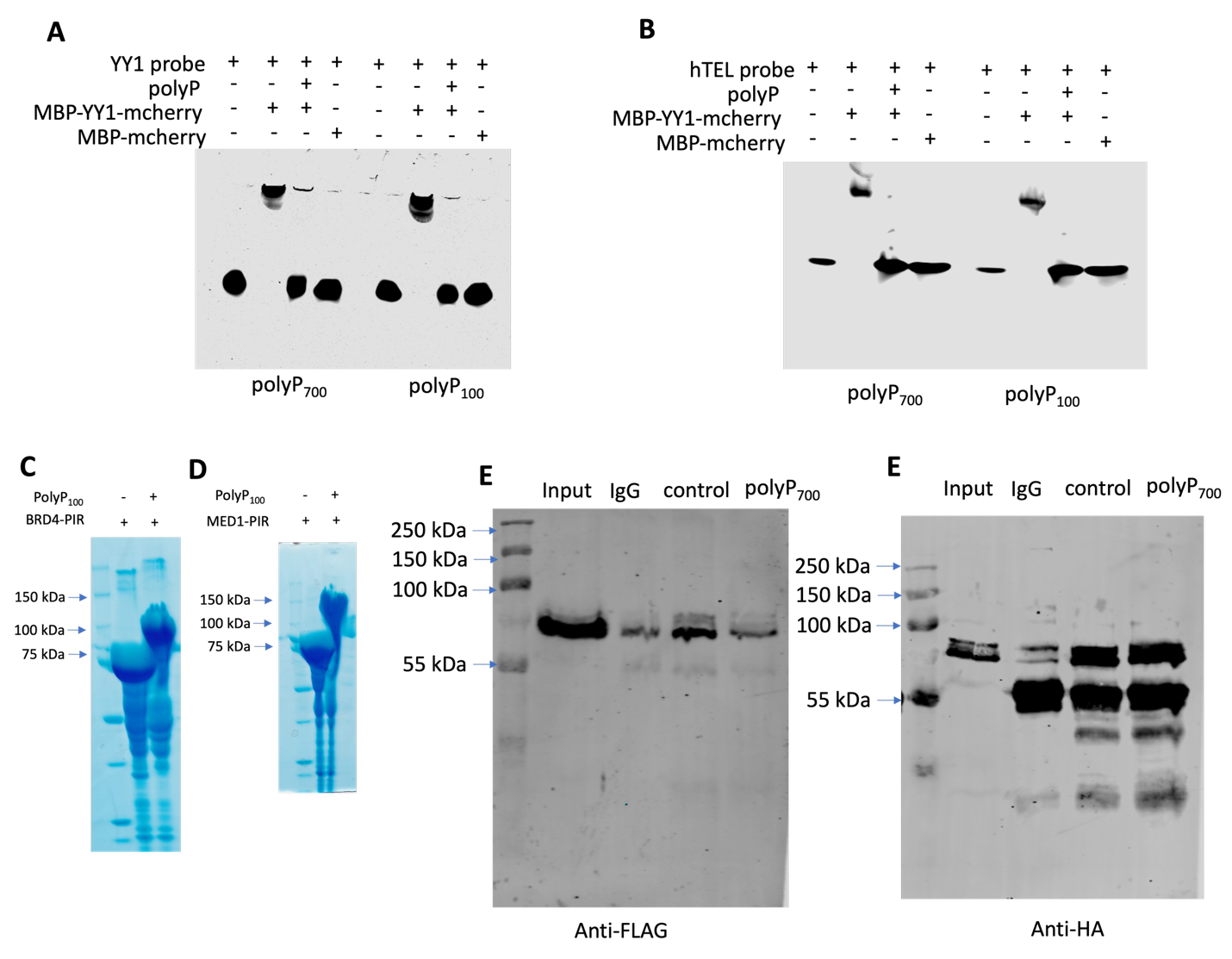
**

**Figure A5.** Original gels or images of Figure 5 and Figure 6. (**A**) Corresponding to Figure 5A and 6E. EMSA results using 4% native gel showing the binding of MBP-mCherry tagged YY1 to IR700Dye-labled YY1 binding motif after polyP_700_ (Corresponding to Figure 5A) or polyP_100_ (Corresponding to Figure 6E) treatment. (**B**) Corresponding to Figure 5E and 6H. EMSA results using 4% native gel showing the binding of MBP-mCherry tagged YY1 to IR700Dye-labled human telomere G4 structure after treatment of polyP_700_ (Corresponding to Figure 5E) or polyP_100_ (Corresponding to Figure 6H). (**C, D**) Corresponding to Figure 6B and 6C. Coomassie-stained NuPAGE analysis showing polyP_100_ modification on MBP-GFP tagged BRD4 PIR (Corresponding to Figure 6B) and MED1 PIR (Corresponding to Figure 6C). (**E**) Corresponding to Figure 6D. Western blot analysis after SDS-PAGE showing the ability of polyP_100_ to impair co-immunoprecipitation of FLAG-tagged YY1 and HA-tagged YY1 proteins from nuclear lysates prepared from transfected cells using antibodies against FLAG or HA.
